# BA08: An open-label, single-arm, non-randomised, phase 2 trial of cisplatin, methotrexate and vinblastine (CMV) for pure squamous cell cancer of the urinary tract

**DOI:** 10.1371/journal.pone.0210785

**Published:** 2019-01-16

**Authors:** Gareth O. Griffiths, Richard A. Cowan, Kenneth M. Grigor, Barbara M. Uscinska, Matthew Sydes, Martin Russell

**Affiliations:** 1 Southampton Clinical Trials Unit, University of Southampton, Southampton, United Kingdom; 2 Christie Hospital, Manchester, United Kingdom; 3 Department of Pathology, Western General Hospital, Edinburgh, United Kingdom; 4 Medical Research Council Clinical Trials Unit, University College London, London, United Kingdom; 5 Institute of Cancer Sciences, University of Glasgow, Glasgow, United Kingdom; Tata Memorial Centre, INDIA

## Abstract

**Background:**

Pure squamous cell carcinoma (SCC) of the urinary tract is rare in the UK and has a poor prognosis compared with transitional cell carcinoma (TCC). Cisplatin based chemotherapy has been shown to be effective in TCC.

**Methods:**

Patients with T3-T4, pelvic relapsed, nodal or metastatic SCC of the urinary tract were recruited into an open-label, single-arm, non-randomised, phase 2 trial evaluating the activity and safety of cisplatin, methotrexate and vinblastine (CMV) chemotherapy. CMV was given as three 21-day cycles of methotrexate 30mg/m^2^ (day 1 & 8), vinblastine 4mg/m^2^ (day 1 & 8) and cisplatin 100mg/m^2^ (day 2).

**Results:**

38 patients were recruited. Overall response was 39% (95% CI 24%, 55%)–13% CR and 26% PR. Median OS was 7.8 months (95% CI 3.4, 12.6) with 39% 1-year survival. Toxicity was acceptable.

**Conclusion:**

CMV is well tolerated and active in patients with pure SCC of the urinary tract.

## Introduction

Pure squamous cell carcinoma (SCC) of the bladder is rare in the UK accounting for 6–7% of all bladder cancer. It has a poor prognosis compared with transitional cell carcinoma (TCC) with a reported 5-year overall survival rate of 2–24%. Squamous cancer invariably invades the bladder wall at presentation and usually causes death by local spread [1–3]. The poor prognosis of these cancers might be related to more advanced primary tumour stage and larger tumour size at presentation compared with transitional cell cancer, although their radiosensitivity, stage for stage, is equivalent [3, 4]. TCC of the urothelium is a chemosensitive disease and cisplatin-based combination chemotherapy has been shown to be active in clinical trials and has become a standard of care for this disease in both the neoadjuvant and metastatic setting [5, 6]. SCC of the bladder generally presents as a locally advanced tumour with distant metastases at the time of presentation being rare. Thus, there might be a greater potential for improving the results in these tumours. However, most studies of chemotherapy in bladder cancer have been restricted to TCC and so the BA08 trial was initiated to investigate cisplatin-based chemotherapy in patients with SCC of the bladder.

## Material and methods

In this open-label, single-arm, non-randomised, phase 2 trial, we evaluated the activity and safety of cisplatin, methotrexate and vinblastine (CMV) chemotherapy in patients with pure squamous cell carcinoma of the urinary tract. The protocol of this Medical Research Council (MRC) BA08 trial was developed in the early 1990s when the UK did not have a centralised ethics approval process (i.e. multi-centre research ethics committees (MRECs)) and before the establishment of the UK Medicines and Healthcare Products Regulatory Agency (MHRA) and EudraCT registration (n.b. it has been registered post completion on http://www.isrctn.com/ISRCTN84356042) however according to regulatory requirements at the time local research ethics committees within each of the recruiting hospitals approved the protocol i.e. Beatson Oncology Centre, Christie Hospital, Clatterbridge Hospital, Middlesex Hospital, Western General, Weston Park, Guys Hospital, Newcastle General, Queen Elizabeth, Norwegian Radium Hospital, Walsgrave Hospital, North Staffordshire Hospital, Oncology centre Warsaw. All clinical investigation was conducted according to the principles expressed in the Declaration of Helsinki.". Although the database was frozen and an abstract accepted and presented at ASCO 2001 it was never written up, this is the first publication of results in a peer reviewed journal. At the time of the trial CMV was the standard of care for TCC. The protocol (S1 File) was reviewed in each recruiting institution by their local research ethics committees according to the national standards at the time (please see in acknowledgements the names of all recruiting hospitals where approval was granted), and informed consent for participation in the study was given by all patients prior to inclusion.

### Eligibility criteria

Eligibility inclusion criteria included patients with pure squamous cell carcinoma of the urinary tract where the following groups were included: i) those with an initial presentation with T3-T4 disease; ii) those with pelvis relapse after radiotherapy or surgery; or iii) those with nodal or metastatic disease. Patients had to have at least one site of disease assessable for response (i.e. an indicator lesion of the primary bladder tumour, other primary tumours in the urinary tract, pelvic relapse and/or nodal/metastases) by clinical examination (e.g. bimanual examination, cystoscopy and TUR biopsy) or imaging (CT scan, MRI and Chest X-rays). Exclusion criteria included patients with transitional cell carcinoma with squamous metaplasia, or other mixed tumours and patient with co-existing illness (e.g. cardiac failure) which may compromise administration of CMV chemotherapy. A full list of inclusion and exclusion criteria is given in S1 Text.

### Treatment

At UK secondary care NHS Trusts all patients received three 21-day cycles of CMV chemotherapy (cisplatin 100mg/m^2^ given on day 2, methotrexate at 30mg/m^2^ and vinblastine at 4mg/m^2^, both given intravenously, on days 1 and 8) from their treating clinician. Folinic acid was given 24h after each methotrexate injection at a dose of 15mg orally or intravenously 6-hourly × 4. Cisplatin was given following a period of intravenous hydration in which at least 1 litre of normal saline was given and was not administered until urine output was measured as equal to or exceeding 100ml h^-1^ for 4h. The administration of cisplatin was followed by 2 litre further hydration with normal saline, with supplementary potassium chloride and magnesium sulphate.

#### Endpoints

The primary endpoint of the study was overall response at 9 weeks (or post-chemotherapy if chemotherapy was stopped) after the commencement of the treatment (i.e. end of 3 cycles). The detail on the definition of response is given in S2 Text and at the time of the trial was the standard assessment of response used in these patients. In summary: the disappearance of all known malignant disease was considered a complete response; an at least a 50% reduction in measured lesions (with no new or progression of existing lesion) was considered a partial response; a less than 50% reduction in measured lesions (with no lesion measured at registration has showing more than a 25% increase) was considered No Change (i.e. stable disease); and a 25% or more increase in the size of one or more lesions, or the appearance of new lesions, was considered progressive disease. Secondary endpoints included treatment compliance, safety and overall survival (OS—time-to-event). Data were collected on paper Case Report Forms (CRFs) by NHS staff at the treating hospital and sent to the MRC Clinical Trials Unit for entry on the clinical trial database (COMPACT).

#### Design and statistical considerations

This trial was conducted using a 3-stage design. In the first stage 16 previously untreated patients were entered and assessed. If no responses were observed the trial would close. If at least one response was observed in these 16 patients then the trial would continue to a second stage to recruit a further 16 previously untreated patients (to a total of 32 patients). If there were fewer than 3 responses in these 32 patients then the trial would close. If the true response rate were 20% the probability of incorrectly rejecting the treatment at the first or second stage would be approximately 5%. If the trial was not stopped at either of these stages, trial entry would continue until a maximum of 45 patients had been entered which would result a standard error of about 7% in the final estimate of the response rate (i.e. a margin of error of approximately 14%). There was no treatment blinding.

The primary endpoint of 9-week response is presented as % and 95% CI [calculated using the normal approximation interval Wald method]. In addition to the primary analysis of 9-week response (post-chemo) in all patients, the responses in those patients with pure SCC will also be calculated. The proportion of patients completing a total of 0,1,2 and 3 cycles of CMV are presented as %s, with reasons listed for non-completion, and median [with 1^st^ quartile [Q_1_] and 3^rd^ quartile [Q_3_], values) doses of each drug in each cycle. Patient toxicities during treatment and also long term are listed. Overall survival is calculated as the time from registration to death from any cause. Patients still alive are censored at the time of last follow up. Overall survival will be presented as a Kaplan-Meier curve with median overall survival (plus 95% Hall-Wellner confidence band) and 1-year rate. Data analysis will be carried out in the SAS statistical package (intention-to-treat) with the Kaplan-Meier curve produced in STATA.

## Results

**Fig 1:**
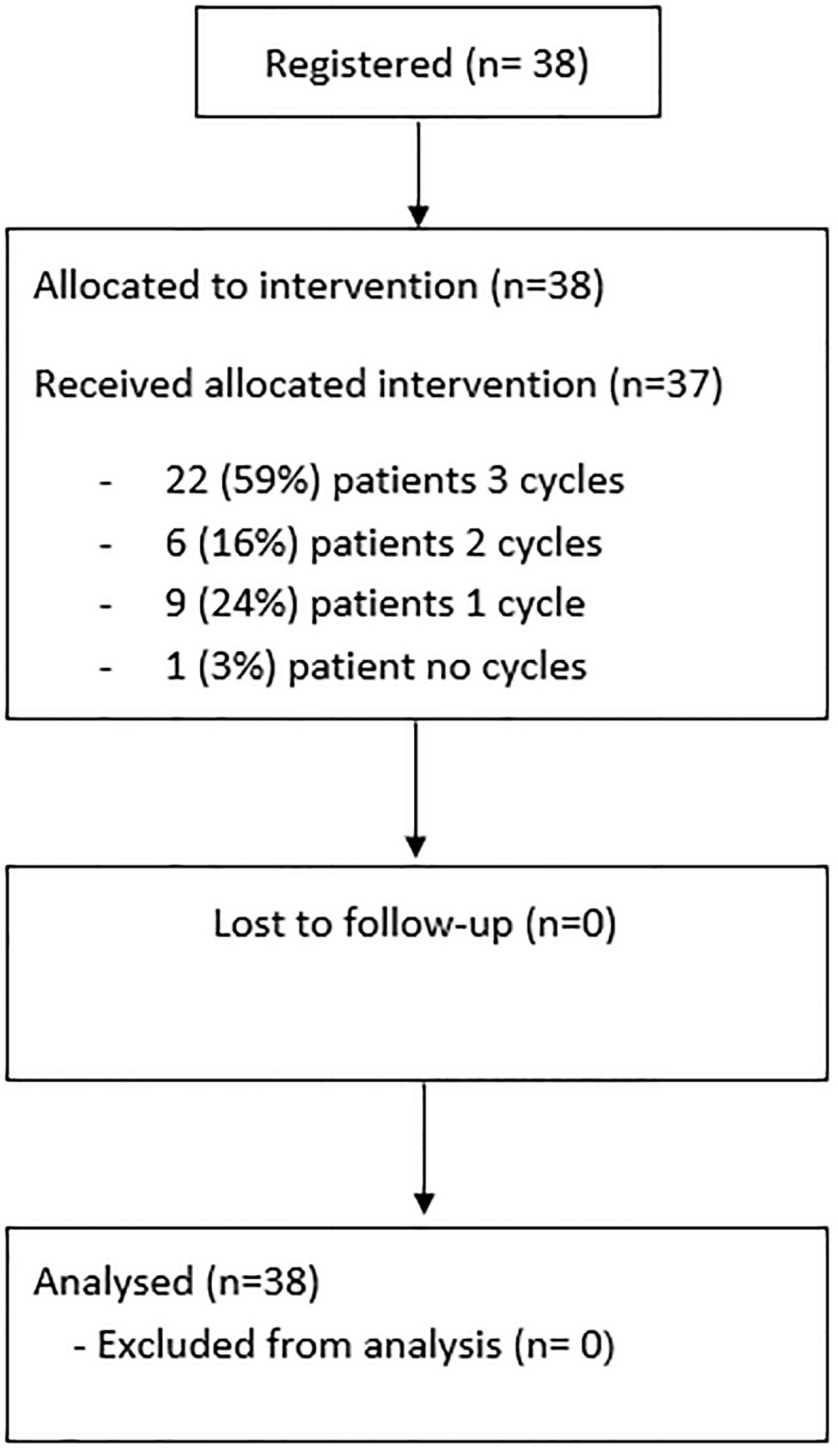
CONSORT diagram.

Between October 1993 and February 1999, 38 patients were recruited from 13 centres within the UK, Norway and Poland. The study progressed through all 3 stages of the design and all 38 patients were included in the analysis. The CONSORT diagram is given in Fig 1 and patient characteristics are reported in Table 1.

**Table 1:** Patient characteristics.

Patient Characteristics		Number (%)
Age	<= 50	12 (32%)
	51–60	10 (26%)
	61–70	9 (24%)
	>= 71	7 (18%)
	Median (Q_1_, Q_3_)	57 (47, 70)
Sex	Male	21 (55%)
	Female	17 (45%)
WHO performance Status	0: Normal activity	15 (40%)
	1: Restricted	14 (37%)
	2: Ambulatory	8 (21%)
	3: Limited self-care	1 (3%)
Primary site of tumour	Bladder	36 (95%)
	Other site	2 (5%)
Previous treatment to primary	None	26 (68%)
	Surgery	8 (21%)
	Radiotherapy	4 (11%)
	Other (e.g. chemo)	0 (0)%
Tumour category	T2	2 (5%)
	T3	25 (66%)
	T4	9 (24%)
	TX	2 (5%)
Lymph nodes involvement	N0	23 (60%)
	N1	4 (10%)
	N2	6 (16%)
	N3	1 (3%)
	N4	1 (3%)
	NX	3 (8%)
Distant metastases	M0	32 (84%)
	M1	5 (13%)
	MX	1 (3%)
Tumour present	Yes	38 (100%)
Tumour type	Squamous cell	36 (95%)
	TCC with squamous	1 (3%)
	Other	1 (3%)
Tumour grade	G2	5 (13%)
	G3	33 (86%)
Muscle present	No	4 (11%)
	Yes	34 (89%)
Muscle (or prostate) invaded	Yes	33 (87%)
	Equivocal	1 (3%)
	Not assessable	4 (11%)
Total		38

For the primary endpoint of 9-week response in all 38 patients, 5 (13%) had a complete response (CR) and 10 (26%) had a partial response (PR), corresponding to an overall response rate of 39% (95% CI 24%, 55%). For the 36 pure SCC patients it was 36% (95% CI 20%,52%), summarised in Table 2.

**Table 2:** Overall outcome.

Outcome	Results	
Primary endpoint—all patients	ALL patients (n = 38)	SCC patients (n = 36)
Post-chemotherapy overall response (%, 95% CI)		
Complete response	5 (13%)	4 (11%)
Partial response	10 (26%)	9 (25%)
No change	9 (24%)	9 (25%)
Progression	6 (16%)	6 (17%)
Not assessable	8 (21%)	8 (22%)
	ORR = 15/38	ORR = 13/36
	39% (95% CI 24%, 55%)	36% (95% CI 20%,52%)

Median post-chemotherapy assessment was at 8.9 weeks (Q_1_, 5.3, Q_3_ 11)

22 out of 38 patients (58%) completed a total of 3 cycles of CMV treatment, with 6 patients (16%) receiving 2 cycles, 9 patients (24%) 1 cycle and 1 patient (3%) no cycles (Table 3). Reasons for not receiving 3 cycles included renal function (n = 4), haematological toxicity (n = 4, 2 with progression), not fit/well (n = 2, 1 with progression), death (n = 2, 1 patient had previous experienced renal toxicity and the other sepsis), sepsis (n = 1), admission to hospice (n = 1, had experienced renal toxicity, vomiting/fainting and progression) and progression for 2 patients. Reasons for delays/reductions of dose in a cycle included protocol violation (n = 8), chest infection (n = 1), renal toxicity (n = 8), haematological toxicity (n = 8) and other reasons (1 femoral embolus, 1 dehydration, 1 mucositis, 1 admin error, 1 tinnitus/headache, 1 abdominal pain). 7 patients reported long term toxicity (i.e. post treatment) due to neuropathy (n = 4, 1 with tinnitus), diarrhoea (n = 1), haematological toxicity (n = 1) and RT telangiectasia (n = 1). These toxicities recorded were similar to those reported in previous transitional cell carcinoma trials using CMV.

**Table 3:** Treatment received.

	Day		Cycle 1	Cycle 2	Cycle 3
**Cisplatin (C)**	2	No. pts with cycle	37	28	22
(100mg/m^2^)		No. pts received C	37^1^	27	22
		Median mg/m^2^	100.0	98.4	98.6
		(Q_1_, Q_3_)	(98.9,100.0)	(50.0, 100.0)	(50.0, 100.0)
**Methotrexate (M)**	1	No. pts with cycle	37	28	22
(30mg/m^2^)		No. pts received M	36	27	22
		Median mg/m^2^	30.0	29.8	29.9
		(Q_1_, Q_3_)	(29.8, 30.3)	(27.8, 30.2)	(29.4, 30.1)
**Methotrexate (M)**	8	No. pts with cycle	37	28	22
(30mg/m^2^)		No. pts received M	26^3^	22^3^	17
		Median mg/m^2^	30.0	29.4	29.8
		(Q_1_, Q_3_)	(29.4, 30.3)	(27.8, 30.2)	(28.9, 30.1)
**Vinblastine (V)**	1	No. pts with cycle	37	28	22
(4mg/m^2^)		No. pts received V	37	27	22
		Median mg/m^2^	4.0	4.0	4.0
		(Q_1_, Q_3_)	(3.9, 4.0)	(3.7, 4.1)	(3.9, 4.0)
**Vinblastine (V)**	8	No. pts with cycle	37	28	22
(4mg/m^2^)		No. pts received V	25	23^3^	17
		Median mg/m^2^	4.0	4.0	4.0
		(Q_1_, Q_3_)	(3.9, 4.0)	(3.7, 4.0)	(3.8, 4.0)

^1^ missing dose data on 1 patient

^3^ missing dose data on 3 patients

**Fig 2:**
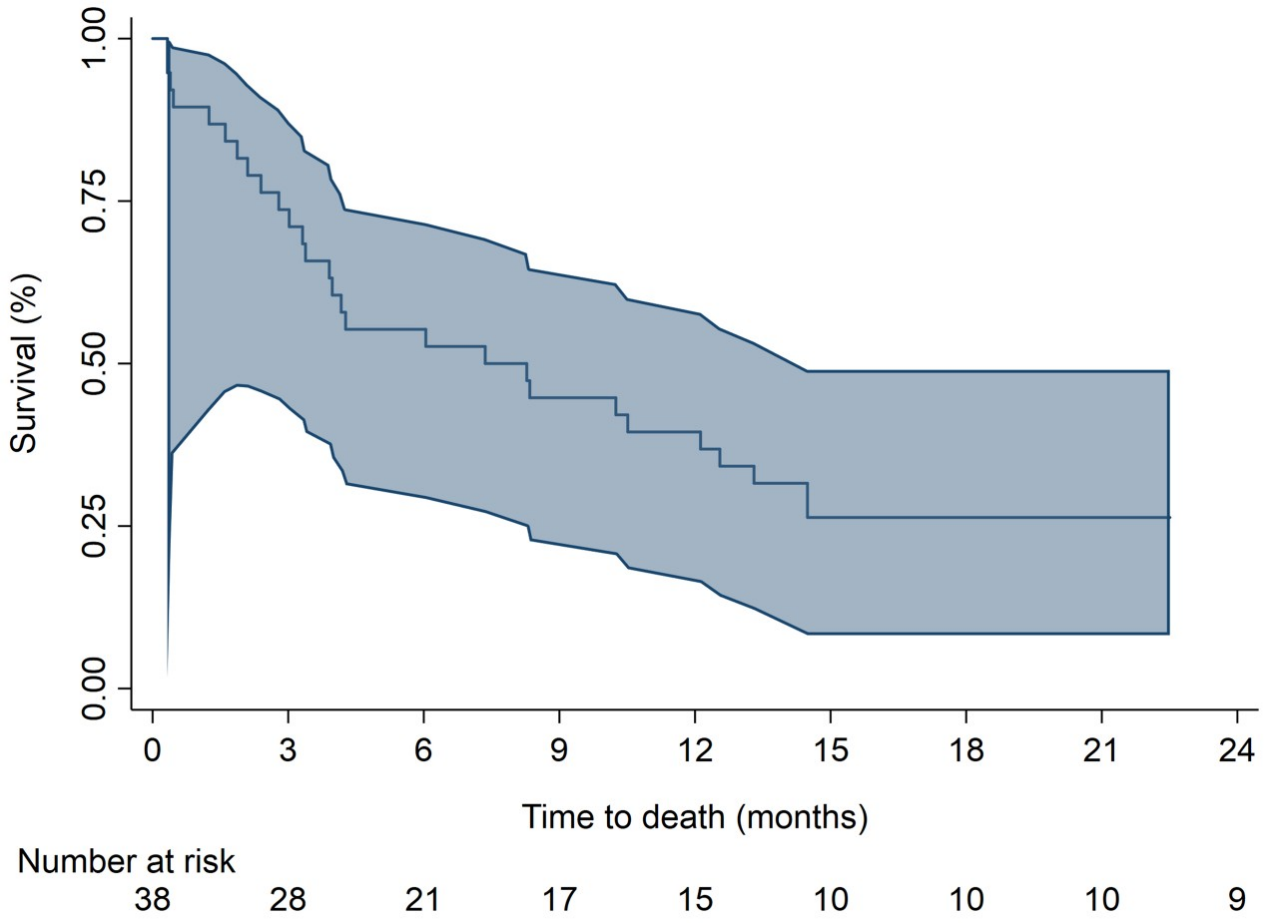
Kaplan-Meier curve of overall survival.

A total of 25 out of 38 patients (66%) received further treatment in their follow-up. 16/38 patients received only RT (either radical and/or palliative), 1/38 received RT and surgery, 4/38 received surgery only, and a total of 4/38 received further chemotherapy (2 only chemotherapy, 2 chemotherapy and surgery). Including all patients, a total of 30 out of 38 have died, 23 deaths (77%) were due to squamous cell carcinoma, 1 (3%) was treatment related and 6 (20%) were due to other reasons. The median survival was 7.8 months (95% CI 3.4, 12.6) with 1-year survival of 39% (95% CI 24.2%, 54.4%) (Fig 2).

## Discussion

Treatment with CMV in SCC appears to be active and safe. If the true response rate were 20% the probability of incorrectly rejecting the treatment at the first or second stage would be approximately 5%. However, at each stage of the design there was sufficiently high activity to continue through all three stages of the design and give an overall response rate of the same magnitude as observed in TCC [6].

There are very few clinical trials involving just SCC of the bladder patients, indeed this being one of the largest ever conducted. Although this trial was conducted over 15 years ago the results are still relevant as little has changed in this patient population with standard of care still being cisplatin based chemotherapy, in fact the UK NICE bladder guidance in 2015 clearly states that “There was insufficient high-quality evidence on which to make specific recommendations” for SCC [7]. Although only a single arm phase II the results are generalisable due to the number of centres involved. In a SCC series, it has been suggested there is an association with an enhanced expression of EGFR and p53 [8] and that markers of apoptosis pathways (e.g. COX-2) might play an important role in its prognosis [9]. With the onset of new targeted treatments (e.g. immunotherapy) there is a need to investigate the impact of new treatment strategies in patients with this cancer. With the development of international partnerships to undertake international clinical trials of rare cancers, such as the International Rare Cancer Initiative (IRCI: a joint initiative between Cancer Research UK, the UK National Institute of Health Research Clinical Research Network [Cancer], the National Cancer Institute (NCI), the European Organisation for Research and Treatment of Cancer (EORTC), the Institut National Du Cancer (INCa), Clinical Oncology Society of Australia (COSA), Japan Clinical Oncology Group (JCOG) and Canadian Cancer Trials Group (CCTG)) there is an opportunity undertake future randomised trials in this cancer. The activity observed in this trial will be used as the control arm rate to improve on with new treatment strategies.

## Conclusions

Treatment with CMV in SCC was well tolerated and was found to be active, appearing to be of a similar magnitude as in TCC. The greatest challenge was recruitment but with more recent opportunities for international collaborations for trials of rare cancers further randomised trials using more modern targeted agents should be considered [10].

## Supporting information

S1 File BA08 trial protocol. (PDF)

S2 File TREND statement. (PDF)

S1 Text Inclusion and exclusion criteria. (PDF)

S2 Text Definition of response. (PDF)
