# Supplementary material for: BA08: An open-label, single-arm, non-randomised, phase 2 trial of cisplatin, methotrexate and vinblastine (CMV) for pure squamous cell cancer of the urinary tract
Source: PLoS One. 2019 Jan 16;14(1):e0210785. doi: 10.1371/journal.pone.0210785 (PMC6334943; doi:10.1371/journal.pone.0210785)
Supplement: S1 File — (PDF) [file pone.0210785.s001.pdf]

MRC
Medical Research Council

BA08

CMV Chemotherapy for Pure Squamous Cell Cancer of the Urinary Tract

Protocol BA08

August 1993

MRC WORKING PARTY IN UROLOGICAL CANCER

SUBGROUP IN ADVANCED BLADDER CANCER

A Phase II Trial of Cisplatin, Methotrexate and Vinblastine (CMV) Chemotherapy for Pure Squamous Cell Cancer of the Urinary Tract

Short Title:CMV for Pure Squamous Cell Cancer of the Urinary Tract

This document is intended to describe a Medical Research Council trial and to provide information about procedures for entering patients. It is not intended that the protocol be used as an aide-memoire or guide for the treatment of other patients. Amendments may be necessary; these will be circulated to known participants in the trial, but centres entering patients for the first time are advised to contact the MRC Cancer Trials Office, Cambridge, to confirm the details of the protocol in their possession.

Clinical problems relating to this trial should be referred to Dr J M Russell, Beatson Oncology Centre, Western Infirmary, Dumbarton Road, Glasgow. (Telephone No. 041 339 8822).

Clinical Co-ordinator
J M Russell

Statistician
M K B Parmar

Data Manager
B M Uscinska

Consultant Oncologist
Beatson Oncology Centre
Western Infirmary
GLASGOW G11 6NT

Tel: 041 339 8822
Fax: 041 337 1712

MRC Cancer Trials Office
5 Shaftesbury Road
CAMBRIDGE
CB2 2BW

Tel: 0223 311110
Fax: 0223 311844

PATIENT REGISTRATION : 0223 322000

LIST OF CONTENTS

SECTION		PAGE
	**Flow Chart for Investigations and Chemotherapy**	
1.0	**Aim of the Trial**	1
2.0	**Background**	1
3.0	**Pre-treatment Assessments**	2
	3.1 Eligibility criteria	2
	3.2 Ineligibility criteria	3
	3.3 Pre-treatment investigations	3
4.0	**Registration**	4
	4.1 Entry of patients	4
	4.2 Information required for registration	4
5.0	**Pathology**	5
6.0	**Summary of Treatment**	5
7.0	**Methods of Drug administration**	6
8.0	**Drug Toxicity**	6
	8.1 Cisplatin	7
	8.2 Methotrexate	7
	8.3 Vinblastine	7
9.0	**Dose Modification**	7
	9.1 Dose modifications for haematological toxicity	8
	9.2 Dose modifications for renal function	8
10.0	**Warning: Drug Interaction**	9
11.0	**Anti-emetic Medication**	9
12.0	**Follow-up**	10
13.0	**Forms and Procedures for Data Collection**	11
14.0	**End-points**	11
15.0	**Statistical Considerations**	12
16.0	**Ethical Considerations**	12
17.0	**Publication**	13
	**References**	13
	**Appendix 1** - Assessment of renal function	14
	**Appendix 2** - Definitions of response	15
	**Appendix 3** - TNM bladder classification	16
	**Appendix 4** - WHO performance status	17
	**Appendix 5** - Toxicity coding	18

FLOW CHART FOR INVESTIGATIONS AND CHEMOTHERAPY

	Day 1	2	8	9	22	23	29	30	43	44	50	51	64
Weeks	0				3				6				9
**Methotrexate** 30mg/m² iv bolus	↓		↓		↓		↓		↓		↓		
**Vinblastine** 4mg/m² iv bolus	↓		↓		↓		↓		↓		↓		
**Folinic acid** 15mg oral or iv 6h x 4		↓		↓		↓		↓		↓		↓	
**Hyc [illegible] for CDDP** N saline 1 litre 2 hourly		↓				↓				↓			
**Cisplatin** 100mg/m² iv infusion		↓				↓				↓			
Physical examination	●				●				●				●
Height	●												
Weight	●				●				●				
Surface area	●				●				●				
WHO	●				●				●				
Full blood count	●		●		●		●		●		●		
GFR	●		●		●		●		●		●		
Alkaline phosphatase	●												○ +
Liver function tests	●												○ +
Liver imaging	○												○ +
Chest x-ray	●				○				○				○ +
Cystoscopy	○												○ +
Bimanual examination	○												○ +
CT scan abdomen/Pelvis	●												○ +

21 days = 1 cycle

Evaluate for response (day 64)

○ If indicated, see page 3
\+ If indicated, see pages 10

1.0 AIM OF TRIAL

The aim of this Phase II trial is to make an approximate assessment of the response rate to CMV chemotherapy in pure squamous cell cancer of the urinary tract.

2.0 BACKGROUND

Pure squamous cell carcinoma of the bladder is rare in the UK (6-7% of all bladder cancer) and has a poor prognosis compared with transitional cell cancer, with reported 5 year overall survival rates of 2 - 24%. Squamous cancers invariably invade the bladder wall at presentation and usually cause death by local spread (1, 2, 3). The poor prognosis of these cancers may be related to more advanced primary tumour stage and larger tumour size at presentation compared with transitional cell cancers, although their radiosensitivity, stage for stage, is equivalent (3, 4).

Cisplatin, methotrexate and vinblastine chemotherapy (CMV) is an effective regime for transitional cell cancer of the urinary bladder (6), and has been evaluated in Phase II studies of metastatic and locally advanced (T3, T4) transitional cell carcinoma of the bladder. The MRC Advanced Bladder Cancer Subgroup has completed a Phase II trial of CMV as primary treatment in T3, T4 TCC with a complete plus partial response rate of 57% after two cycles of chemotherapy (5). Chemotherapy alone can therefore induce complete or partial response of the primary tumour in invasive TCC of the bladder. Consequently, neo-adjuvant chemotherapy has been proposed as a way of improving the results of local definitive treatment in these cancers, and the MRC together with the EORTC and a number of other organisations are currently conducting a randomised trial of this therapy in locally advanced TCC of the bladder.

Squamous cell cancer of the bladder generally presents as a locally advanced tumour - distant metastases at the time of presentation are rare. Thus, there may be a greater potential for improving the results in these tumours. However, most studies of chemotherapy in bladder cancer have been restricted to transitional cell cancer (7,8) with little available data on squamous cancer.

Chemotherapy regimes containing cisplatin and methotrexate have been used for squamous cancers at other sites, e.g. cancer of the cervix (9) and in head and neck cancers. In head and neck cancer, cisplatin containing regimes have yielded response rates of 60% when used prior to radiotherapy (10). Cisplatin, methotrexate and vinblastine may therefore be an effective regime in squamous cancer of the bladder. The MRC therefore propose a single arm Phase II trial of CMV in locally advanced, recurrent, and metastatic pure squamous cancer of the bladder and other sites in the urinary tract.

3.0 PRE-TREATMENT ASSESSMENTS

3.1 Eligibility Criteria

1) Any patient with **pure squamous cell carcinoma of the urinary tract** in one of the following groups:

   i. initial presentation with T3 - T4 disease
   ii. pelvic relapse after radiotherapy or surgery
   iii. nodal or metastatic disease.

2) At least **one site of disease must be assessible** for response by clinical examination or imaging. One or more of the sites listed below can be used to assess response.

   i.a ***Primary Bladder Tumour***
   Satisfactory measurements of the primary tumour if present and measurable, must have been made by bimanual examination **after** transurethral resection (TUR).

   i.b ***Other Primary Tumours in the Urinary Tract***
   Satisfactory measurements of the primary tumour in the urinary tract, if present and measurable, must have been made by clinical examination or appropriate imaging.

   ii. ***Pelvic Relapse after Radiotherapy or Surgery***
   At least one indicator lesion must be measurable by clinical examination or appropriate imaging.

   iii. ***Nodal or Metastatic Disease***
   At least one indicator lesion must be measurable by clinical examination or appropriate imaging.

   Bone metastases cannot be used as an indicator lesion.

3) Calculated Glomerular filtration rate 50ml/min, using the formula of Cockcroft and Gault (Appendix 1). In patients with impaired renal function secondary to ureteric obstruction this may be relieved by ureteric stents or nephrostomies, and if the renal function then recovers the patient will be eligible.

4) WBC > 3.5 x 10^9/l and platelet count > 100 x 10^9/l.

5) No previous systemic treatment with chemotherapy.

6) No concomitant or previous malignancy other than BCC of skin or carcinoma-in-situ of the cervix.

7) Patients should be fit to tolerate CMV.

3.2 Ineligibility criteria

i. Patients with transitional cell carcinoma with squamous metaplasia, or other mixed tumours are ineligible.

ii. Co-existing illness (e.g. cardiac failure) which may compromise administration of CMV chemotherapy.

3.3 Pre-treatment investigations

i. History and physical examination to detect areas of metastatic disease and assess fitness to receive cisplatin containing chemotherapy.

ii. If the indicator lesion is a primary bladder tumour then this must be measured by bimanual examination under anaesthesia **after** TUR.

iii. If the indicator lesion is a primary tumour elsewhere in the urinary tract, pelvic relapse, nodal or distant metastatic disease then it must be measured by appropriate clinical or radiological examination.

iv. Height, weight, surface area and WHO performance status (see appendix 4).

v. Haemoglobin, white blood cell count and platelet count.

vi. Serum creatinine. Glomerular filtration rate should be estimated by calculation (see Appendix 1).

vii. Liver function tests. Liver imaging should be performed if liver function tests are abnormal or if patient has symptoms suggesting liver metastases.

viii. Alkaline phosphatase. Isotope bone scan and liver imaging should be performed if alkaline phosphatase is raised or patient's symptoms suggest bone or liver involvement respectively.

ix. Chest x-ray.

x. CT scan of abdomen and pelvis.

4.0 REGISTRATION

4.1 Entry of patients

i. All patients should be registered by telephoning the MRC Cancer Trials Office **(0223 322000).**

ii. The participant will be asked to confirm the eligibility criteria (section 3.1) **(Note, the patient will only be registered if the measurements of at least one indicator lesion are given over the telephone)**

iii. Following registration of a patient, the Registration Checklist should be sent immediately to the Trials Office.

4.2 Information required for registration

1) Name of patient

2) Name of hospital and hospital number

3) Name of responsible physician

4) Name of responsible pathologist, pathologist's hospital and biopsy reference number

5) Date of birth

6) WHO performance status (Appendix 4)

7) Sex

8) Previous treatment ~~to primary~~

9) Primary site of tumour

~~10) TNM Stage of primary bladder tumour (if applicable)~~

11) Measurable disease status

The answer to at least one question must be YES

i. ***Measurable primary tumour at presentation (T3-T4)***

If YES, size of indicator lesion (cm) and method of measurement

ii. ***Measurable pelvic relapse after radiotherapy or surgery***

If YES, size of indicator lesion (cm) and method of measurement

iii. ***Measurable nodal or metastatic disease***

If YES, size of indicator lesion (cm) and method of measurement

12) Sites of non-measurable disease, if any.

5.0 PATHOLOGY

Shortly after registration of a patient, the pathologist of your institution will receive a request from the Review Pathologist for one unstained section from each block of tissue. These should be sent to the Review Pathologist without delay for confirmation of the diagnosis of pure squamous cell carcinoma and the presence of muscle invasion.

Similarly, for patients classified as having a complete response in the bladder following chemotherapy, slides from the resection biopsy or bladder specimen will be requested by the Review Pathologist.

6.0 SUMMARY OF TREATMENT

All patients are to receive three cycles of CMV chemotherapy and then should be assessed for response. If there is deterioration of symptoms or clinical progression of disease before 3 cycles are completed, chemotherapy will be discontinued and patients will be reassessed by clinical examination and imaging studies to confirm progression.

CMV chemotherapy is given on days 1, 2 and 8 of a 21 day cycle.
On days 1 and 8 methotrexate (30mg/m^2) iv and vinblastine (4mg/m^2) iv, are given and on day 2 cisplatin (100mg/m^2) iv.

Folinic acid 15mg orally/iv is given six hourly x 4 starting 24 hours after injection of methotrexate (i.e. on days 2 and 9).

The above comprises one treatment cycle - a new cycle starts on day 22, subject to blood counts and renal function. Patients will remain in hospital for one or two nights.

7.0 METHODS OF DRUG ADMINISTRATION

7.1 *Day 1*

Methotrexate	$30 mg/m^2$	By iv bolus, given after FBC and GFR have been checked.
Vinblastine	$4 mg/m^2$	Fluid intake of 2 litres during the day is recommended, but special hydration or the use of mannitol are not required.

7.2 *Day 2*

Cisplatin	$100 mg/m^2$	See *Hydration and Cisplatin*
Folinic Acid	15mg oral or iv six hourly x 4 to be started 24 hours after MTX administration on day 1.	

HYDRATION AND CISPLATIN

Commence hydration for cisplatin on day two. Give N saline 1 litre two hourly and monitor urine output.

When patient has received at least 1 litre of N saline iv and has passed more than 100 ml of urine/hour for at least 4 hours give iv cisplatin.

During the following 16 hours, the patient should be given 2 litres of saline to obtain and maintain optimal diuresis which is 100 ml/hour over 24 hours. This should be checked after six hours and if the urine output falls below 100 ml/hour despite adequate hydration, either mannitol (20-25% 100 ml) or low dose frusemide (10-20 mg) should be given.

In case of diarrhoea and/or nausea and vomiting, the volume of fluid lost should be replaced by additional iv infusion.

Potassium and magnesium should be supplemented: 20 m. equiv. KCl+1G of $MgSO_4$/litre of saline post-hydration is usually sufficient.

7.3 *Day 8*

Methotrexate	$30 mg/m^2$	By iv bolus, given after FBC **and creatinine*** (with calculated GFR) have been checked.
Vinblastine	$4 mg/m^2$	Fluid intake of 2 litres per day for days 8+9 is recommended, but special hydration or the use of mannitol are not required.

* this is **mandatory**

7.4 *Day 9*

Folinic Acid	15mg oral or iv six hourly x 4 to be started 24 hours after MTX administration on day 8.

8.0 DRUG TOXICITY

8.1 Cisplatin

The most important side effects are nausea and vomiting which can be minimised by routine anti-emetic therapy. Renal function disturbances can be largely prevented by adequate hydration and diuresis. Myelosuppression is generally mild to moderate and reaches its nadir between days 10 - 14. Tinnitus and high pitched hearing loss may occur, as may peripheral neuropathy. In the event of infectious complications, aminoglycosides (e.g. gentamicin) should be avoided because of possible toxicity to the kidney.

8.2 Methotrexate

Although methotrexate can usually be given safely with only minor subjective and objective toxicity, it has to be recognised that severe bone marrow and mucosal toxicity can occur if the drug is excreted slowly by the kidney. It is therefore of paramount importance that methotrexate is not given in the presence of impaired renal function, regardless of whether this is caused by parenchymal function loss or by obstructive uropathy. **The clinician must know the value of the GFR calculated from serum creatinine (see Appendix 1) immediately before the administration of methotrexate and must use the guide-lines for dose reductions and delay as described in sections 9.1 and 9.2.** Preventive measures during methotrexate therapy are a fluid intake of 4 litres during the first 48 hours following methotrexate administration and good oral hygiene including toothbrushing with a smooth brush and regular mouth washes. Aspirin-containing analgesics as well as any non-steroidal anti-inflammatory drug, sulphonamides and high doses of vitamin C should be avoided during and for at least 3 days following methotrexate administration because these drugs slow down its excretion by the kidney.

8.3 Vinblastine

Vinblastine is a myelosuppressive drug which can also cause mild peripheral neuropathy. Constipation and alopecia may occur. This drug is a vesicant and care should be taken to avoid extravasation.

9.0 DOSE MODIFICATION

Protocol chemotherapy may have to be modified if toxicity arises. Dose modifications will be based upon the following investigations which are performed prior to administration. Doses may have to be modified according to renal and haematological toxicity on the day in question or during the preceding cycles(s). **If haematological and renal toxicity co-exist** and more than one dose reduction applies, calculate each separately and then always employ the lowest dose applicable.

9.1 Dose modifications for haematological toxicity

Reductions apply to all drugs given on days 1, 2 and 8.

	Cisplatin	Methotrexate	Vinblastine
**WBC > 3.5 and platelets > 100**	100%	100%	100%
**WBC 3.0 - 3.5 and platelets > 100**	100%	75%	75%
**WBC 2.5 - 2.9 and platelets > 100**	100%	50%	50%

WBC < 2.5 or platelets < 100

1) DAY 1 Delay treatment for up to two weeks (weekly FBC). If WBC still <2.5 or platelets < 100 discontinue therapy.

2) DAY 8. Omit chemotherapy. Recommence on DAY 22, adjusting doses according to FBC and renal function at that time.

9.2 Dose Modifications for renal function

Creatinine clearance should be calculated on days 1 and 8 using the Cockcroft formula (see appendix 1).

Reductions apply to methotrexate (days 1 and 8) and cisplatin (day 2).

Note the dose of vinblastine should not be reduced for renal toxicity.

Glomerular Filtration Rate	Cisplatin	Methotrexate	Vinblastine
**≥60ml/min**	100%	100%	100%
**45 - 60ml/min**	50%	100%	100%
**≤45ml/min**	0%	0%	100%

1) If GFR is ≤45ml/min on DAY 1, repeat GFR after one week and consider checking radio-isotope clearance. If no recovery, stop chemotherapy.

2) If GFR ≤45ml/min on DAY 8, omit methotrexate, but treat with i.v. vinblastine according to FBC (see above).

All patients should receive full dose cisplatin, methotrexate and vinblastine on days 1 and 2 of cycle 1 (**even if their GFR at entry is between 50 and 60ml/min**) For day 8 of cycle 1 and all of cycles 2 and 3 the dose reductions described above still apply.

10.0 WARNING: DRUG INTERACTION

Aspirin containing analgesics, non-steroidal anti-inflammatories, sulphonamides or high dose vitamin C should not be given for at least three days following protocol treatment (see Section 6.2). Aminoglycosides may interact with cisplatin (see Section 6.1). If frusemide is required, low doses only (10 - 20mg) should be given.

11.0 ANTI-EMETIC MEDICATION

Nausea and vomiting should be minimised by the regular use of anti-emetic drugs. If no local regimen is preferred the following two regimens are recommended.

i) Metoclopramide 3mg x body weight (kg) by 15 min infusion commencing 30 min prior to cisplatin. Metoclopramide may cause extra pyramidal symptoms such as oculogyric crisis for which we would recommend procyclidine hydrochloride (Kemadrin) which should be immediately available and 5-10mg administered iv as necessary.

Lorazepam 2mg iv stat<br>Dexamethasone 8mg iv by 5 min injection or 15 min infusion	) immediately prior to cisplatin infusion

ii)

Dexamethasone 8mg iv by 5 min injection or 15 min infusion<br>Ondansetron 8mg iv	) immediately prior to cisplatin infusion

12.0 FOLLOW-UP

12.1 Follow-up investigations

i. ~~During chemotherapy~~ at ~~each visit for a drug~~ injection:
Full blood count ~~and calculated creatinine clearance.~~

ii. On day 1 of each treatment course:
Physical examination, weight and chest x-ray (if initially positive).

12.2 ~~Post-chemotherapy assessment~~

~~After 3 treatment cycles (i.e. at/or about day 63)~~ patients will be re-evaluated by physical examination and appropriate imaging techniques, repeating all investigations documented as abnormal at the commencement of chemotherapy. In particular measurements of the indicator lesion specified at the time of registration must be made.

Complete and partial responses ~~after completing 3 cycles of chemotherapy~~ will be recorded (as defined in Appendix 2). Patients failing to complete ~~3 cycles of~~ chemotherapy should be assessed for response at the time of withdrawal from protocol treatment.

i.a ***Primary bladder tumour***

Bimanual examination, cystoscopy and TUR biopsy under general anaesthesia will provide the basis for assessment of the primary tumour. **This should be carried out by the urologist who performed the initial assessment. Note that this is an assessment of the tumour response to chemotherapy alone, and therefore the bimanual examination should be carried out <u>before</u> TUR** (this is different from the situation at entry to trial). If there appears to be a complete response on bimanual examination and cystoscopy, it is essential to take a thorough TUR biopsy at the site of the previous tumour.

i.b ***Other primary tumours in the urinary tract***

The method of measurement should be the same as the methods used at the time of registration.

ii. ***Pelvic relapse***

The method of measurement should be the same as the methods used at the time of registration.

iii. ***Nodal or metastatic***

The method of measurement should be the same as the methods used at the time of registration.

12.3 **After completion of protocol chemotherapy**

i. Following chemotherapy, further treatment and follow-up will be at the discretion of the responsible clinician. If the pre-treatment staging indicates a local (T3) tumour with no metastases, further definitive surgical or radiation treatment should be considered.

ii. For the purpose of the trial, follow-up forms will be completed at 6-monthly intervals to record disease status, long term toxicity and survival.

13.0 FORMS AND PROCEDURES FOR DATA COLLECTION

Following telephone registration of a patient the self-duplicating copy of the checklist should be sent to the Trials Office.

Thereafter forms should be sent to the Trials Office on the following occasions:

**Time**	**Forms to Send**
On Completion of Chemotherapy (3 months after registration)	Chemotherapy Form<br>Post-Chemotherapy Assessment Form
At 6 months	First Follow-up Form
6-monthly follow-up thereafter	Follow-up Form

14.0 END-POINTS

14.1 The principal end-point of this Phase II trial is partial response as defined in Appendix 2 and assessed at the evaluation 9 weeks after commencement of the treatment. A secondary analysis of the response of the primary tumour will also be made. A further analysis will be made to assess whether prior treatment has any effect on response to CMV chemotherapy.

14.2 Treatment toxicity arising from the chemotherapy will be recorded.

14.3 Survival.

15.0 STATISTICAL CONSIDERATIONS

15.1 This is a rare tumour and it is anticipated that 10-15 patients will be entered into this trial annually.

15.2 A maximum of 45 evaluable patients will be entered into this trial leading to a standard error of about 7% in the final estimate of the response rate. This trial will be conducted using a 3-stage design. In the first stage 16 previously untreated patients will be entered and assessed. If no responses are observed the trial will close. If at least one response is observed in these 16 patients then the trial will continue to a second stage to recruit a further 16 previously untreated patients (to a total of 32 patients). If there are fewer than 3 responses in these 32 patients then the trial will close. If the true response rate is 20% the probability of incorrectly rejecting the treatment at the first or second stage is approximately 5%. If the trial is not stopped at either of these stages, trial entry will continue until a maximum of 45 patients have been entered.

16.0 ETHICAL CONSIDERATIONS

16.1 The trial protocol must be approved by the ethical committee before patients are entered. The patient's consent to participate in the trial should be obtained after a full explanation has been given of the treatment options, including conventional and generally accepted methods of treatment, and the manner of treatment allocation.

16.2 The right of the patient to refuse to participate without giving reasons must be respected. After the patient has entered the trial the clinician must remain free to give alternative treatment to that specified in the protocol at any stage if he/she feels it to be in the patient's best interests, but the reasons for doing so should be recorded and the patient will need to remain in the trial for the purpose of follow-up and data analysis according to the treatment option to which he/she has been allocated. Similarly, the patient must remain free to withdraw at any time from protocol treatment without giving reasons and without prejudicing his/her further treatment.

16.3. A statement of MRC policy on ethical considerations in the clinical trial of cancer therapy, including the question of informed consent, is available from the Cancer Therapy Committee Secretariat, Medical Research Council, 20 Park Crescent, London W1N 4AL, and may be used to give guidance to participating investigators and to accompany applications to the local ethical committee.

17.0 PUBLICATION

The result from different centres will be analysed together and published as soon as possible. Individual clinicians must not publish either data concerning their patients which are directly relevant to the questions posed by the trial, or interim analyses of the collective data, until such time as the Working Party have published their report of the trial.

REFERENCES

1. Johnson, DE, Schoenwald MC, Ayala EG & Miller LS. Squamous cell carcinoma of bladder. J.Urol. ***115***: 542-544, 1976.

2. Jones MA, Bloom HJG, Williams G, Trott PA & Wallace, D M. The management of squamous cell carcinoma of the bladder. B.J. Urol. ***52***: 511-514, 1980.

3. Costello AJ, Tiptaft RC, England HR & Blandy JP. Squamous cell carcinoma of bladder. Urology ***23***: 234-236, 1984.

4. Quilty, PM & Duncan W. Radiotherapy for squamous carcinoma of the urinary bladder. Int. J.Rad.Oncol.Biol.Phys. ***12***: 861-865, 1986.

5. Fossa, SD et al. Initial combination chemotherapy with cisplatin, methotrexate and vinblastine in locally advanced transitional cell carcinoma - response rate and pitfalls. Br.J.Urol. ***70***: 161-168, 1992.

6. Harker GH, Meyers FJ, Freiha FS et al. Cisplatin, methotrexate and vinblastine (CMV): an effective chemotherapy regimen for metastatic transitional cell carcinoma of the urinary tract. A Northern Californian Oncology Group Study. J.Clin.Oncol. 3: 1463-1470, 1985.

7. Raghavan D et al. Initial intravenous cisplatinum therapy : improved management for invasive high risk bladder cancer? J.Urol. ***133***: 403-407, 1985.

8. Sternberg CN et al. Preliminary results of M- VAC for transitional cell carcinoma of the urothelium. J.Urol. ***133***: 403-407, 1985.

9. Rosenthal CJ, Khulpateea, N, Boyce J, Mehrota S & Tamarin S. Effective chemotherapy for advanced carcinoma of the cervix with bleomycin, cisplatin, vincristine and methotrexate. Cancer ***52***: 2025-2030, 1983.

10. Tannock I, Cummings B, Sorrenti V and the ENT Group. Combined chemotherapy used prior to radiation therapy for locally advanced squamous cell carcinoma of the head and neck. Cancer Treat.Rep. ***66***: 1421-1424, 1982.

APPENDIX 1

ASSESSMENT OF RENAL FUNCTION

CMV chemotherapy as used in this protocol requires accurate measurements of the glomerular filtration rate on Day 1 and 8 of each treatment cycle.

Individual centres may have preferred techniques of calculating GFR but the following formula is recommended as it relates GFR to body weight, age, sex and the serum creatinine. It is considered adequate for the purposes of this trial and is quicker than 24 hour urine collection or radio-isotope techniques. If GFR is found to be less than 45 ml/min on Day 1, precluding therapy with cisplatin, clinicians may prefer to check GFR using a radio-isotope technique.

COCKCROFT AND GAULT FORMULA

If creatinine measured in mol/l:

GFR (ml/min) =

males:

$$\frac{1.23 \times (140 - \text{age}) \times \text{weight (kg)}}{\text{serum creatinine (mol/l)}}$$

females:

$$\frac{1.05 \times (140 - \text{age}) \times \text{weight (kg)}}{\text{serum creatinine (mol/l)}}$$

If creatinine measured in mg%

GFR (ml/min) =

males:

$$\frac{(140 - \text{age}) \times \text{weight (kg)}}{72 \times \text{creatinine (mg\%)}}$$

females:

$$\frac{(140 - \text{age}) \times \text{weight (kg)} \times 0.85}{72 \times \text{creatinine (mg\%)}}$$

APPENDIX 2

DEFINITIONS OF RESPONSE

Complete Response (CR)

The disappearance of all known malignant disease.

Primary Bladder Tumour

If the initial primary tumour was in the bladder, and cystectomy has not been performed, an assessment should be made of this primary tumour.

If the response of the primary bladder tumour is classified as complete response (CR) on bimanual examination and cystoscopy, a deep resection biopsy should be performed at the site of the original tumour and recorded as biopsy positive or negative, i.e.:

CR (B-)	Complete response on cystoscopic and bimanual examination. Biopsy negative.
CR (B+)	Complete response on cystoscopic and bimanual examination. Biopsy positive.
CR(Bo)	Complete response on cystoscopic and bimanual examination. Biopsy not done.

Partial Response (PR)

At least 50% reduction of the sum of the products of the two largest perpendicular diameters of all lesions measured at registration. In addition there can be no appearance of new lesions nor progression of any lesion.

No Change (NC)

Less than 50% reduction of the sum of the products of the two largest perpendicular diameters of all lesions measured at registration and no lesion measured at registration has shown a 25% increase in size.

Progressive Disease

A 25% or more increase in the size of one or more lesions measured at registration, or the appearance of new lesions.

APPENDIX 3
TNM CLASSIFICATION (1982)

BLADDER (ICD-O 188)

RULES FOR CLASSIFICATION

The classification applies only to epithelial tumours.

Papilloma is excluded but such cases should be listed under the category "G0".

Papillary non-invasive carcinoma should be listed under the category Ta. There should be histological or cytological verification of the disease. Any unconfirmed cases must be reported separately.

The following are the minimum requirements for assessment of the T, N and M categories. If these can not be met the symbol TX, NX, or MX will be used.

T categories: Clinical examination, urography, cystoscopy, bimanual examination under anaesthesia and biopsy or transurethral resection of the tumour (if indicated) prior to definitive treatment

N categories: Clinical examination and radiography including lymphography and urography.

M categories: Clinical examination and radiography. In the more advanced primary tumours or when clinical suspicion warrants radiographic or isotope studies are recommended.

REGIONAL AND JUXTA-REGIONAL LYMPH NODES

The Regional Lymph Nodes are the pelvic nodes below the bifurcation of the common iliac arteries. The Juxta-regional Lymph Nodes are the inguinal nodes, the common iliac nodes and the para-aortic nodes.

TNM PRE-TREATMENT CLINICAL CLASSIFICATION

T - Primary Tumour

- Tis Pre-invasive carcinoma (carcinoma in situ): "Flat tumour".
- Ta Papillary non-invasive carcinoma
- T0 No evidence of primary tumour.
- T1 On bimanual examination a freely mobile mass may be felt: this should not be felt after complete transurethral resection of the lesion and/or microscopically, the tumour does not invade beyond the lamina propria
- T2 On bimanual examination there is induration of the bladder wall which is mobile. There is no residual induration after complete transurethral resection of the lesion and/or There is microscopic invasion of superficial muscle.
- T3 On bimanual examination induration or a nodular mobile mass is palpable in the bladder wall which pe rsists after transurethral resection of the exophytic portion of the lesion and/or There is a microscopic invasion of deep muscle or of extension through the bladder wall.
- T3a Invasion of deep muscle
- T3b Invasion through the bladder wall
- T4 Tumour fixed or extending to neighbouring structures and/or There is microscopic evidence of such involvement.
- T4a Tumour infiltrating the prostrate, uterus or vagina
- T4b Tumour fixed to the pelvic wall and/or abdominal wall
- Note: The suffix (m) may be added to the appropriate T category to indicate multiple tumours, eg T2(m).
- TX The minimum requirements to assess the primary tumour cannot be met.

N - Regional and Juxta-regional Lymph Nodes

- N0 No evidence of regional lymph node involvement.
- N1 Evidence of involvement of a single homolateral regional lymph node
- N2 Evidence of involvement of contralateral or bilateral or multiple regional lymph nodes
- N3 Evidence of involvement of fixed regional lymph nodes (there is a fixed mass on the pelvic wall with a free space between this and the tumour)
- N4 Evidence of involvement of juxta-regional lymph nodes
- NX The minimum requirements to assess the regional and/or juxta-regional lymph nodes cannot be met.

M - Distant Metastases

- M0 No evidence of distant metastases
- M1 Evidence of distant metastases
- MX The minimum requirements to assess the presence of distant metastases cannot be met.

p.TNM POST-SURGICAL HISTOPATHOLOGICAL CLASSIFICATION

pT - Primary Tumour

- pTis Pre-invasive carcinoma (carcinoma in situ)
- pTa Papillary non-invasive carcinoma
- pT0 No evidence of tumour found on histological examination of specimen
- pT1 Tumour not extending beyond the lamina propria
- pT2 Tumour with invasion of superficial muscle (not more than half way through muscle coat)
- pT3 Tumour with invasion of deep muscle (more than way through muscle coat) or with invasion of perivesical tissue
- pT4 Tumour with invasion of prostate or other extravesical structures
- pTX The extent of invasion can not be assessed

G - Histopathological Grading

- "G0" Papilloma, ie. no evidence of anaplasia
- G High degree of differentiation
- G2 Medium degree of differentiation
- G3 Low degree of differentiation or undifferented
- GX Grade can not be assessed

L - Invasion of Lymphatics

- L0 No lymphatic invasion
- L1 Evidence of invasion of superficial lymphatics
- L2 Evidence of invasion of deep lymphatics
- LX Lymphatic invasion can not be assessed

APPENDIX 4/ 3

WHO PERFORMANCE STATUS

Grade	Performance Status
0	Able to carry out normal activity without restriction.
1	Restricted in physically strenuous activity but ambulatory and able to carry out light work.
2	Ambulatory and capable of all self-care but unable to carry out any work; up and about more than 50% of waking hours.
3	Capable of only limited self-care; confined to bed or chair more than 50% of waking hours.
4	Completely disabled; cannot carry out any self-care, totally confined to bed or chair.

APPENDIX ~~5~~ 4,
TOXICITY CODING

	GRADE 0	GRADE 1	GRADE 2	GRADE 3	GRADE 4
Leucopenia	$\geq 4.0X10^{9}/l$	$3.0\text{-}3.9x10^{9}/l$	$2.0x2.9x10^{9}/l$	$1.0\text{-}1.9x10^{9}/l$	$<1.0x10^{9}/l$
Thrombocytopenia	$\geq 100x10^{9}/l$	$75\text{-}99x10^{9}/l$	$50\text{-}74x10^{9}/l$	$25\text{-}49x10^{9}/l$	$<25x10^{9}/l$
Nausea/vomiting	None	Nausea	Transient vomiting	Vomiting requiring therapy	Intractable vomiting
Mucosal/Oral	No change	Soreness/ erythema	Erythema ulcers: can eat solids	Ulcers: requires liquid diet only	Alimentation not possible
Diarrhoea	None	Transient $\leq$ 2 days	Tolerable but > 2 days	Intolerable requiring therapy	Haemorrhagic dehydration
Renal<br>Blood urea or creatinine	≤1.25xN*	1.26-2.5xN*	2.6-5xN*	5.1-10xN*	>10xN*
Liver					
Bilirubin	≤1.25xN*	1.26-2.5xN*	2.6-5xN*	5.1-10xN*	>10xN*
SGOT/SGPT	≤1.25xN*	1.26-2.5xN*	2.6-5xN*	5.1-10xN*	>10xN*
Alkaline phosphate	≤1.25xN*	1.26-2.5xN*	2.6-5xN*	5.1-10xN*	>10xN*
Hematuria	No change	Microscopic	Gross	Gross + clots	Obstructive uropathy
Skin	No change	Erythema	Dry desquamation vesiculation pruritus	Moist desquamation ulceration	Exfoliative dermatitis; necrosis requiring surgical intervention
Neurotoxicity	None	Paraesthesiae and/or decreased tendon reflexes	Severe paraesthesiae and/or mild weakness	Intolerable paraesthesiae and/or marked motor loss	Paralysis
Cardiotoxicity					
Rhythm	No change	Sinus tachycardia >110 at rest	Unifocal PVC arrhythmia	Multifocal PVC	Venticular tachycardia
Function	No change	Asymptomatic but abnormal cardiac sign	Transient symptomatic dysfunction: no therapy required	Symptomatic atrial dysfunction responsive to therapy	Symptomatic dysfunction non-responsive to therapy
Pericarditis	No change	Asymptomatic effusion	Symptomatic: no tap required	Tamponade: tap required	Tamponade: surgery required
Ototoxicity	No change	Slight hearing loss	Moderate hearing loss	Major hearing loss	Complete hearing loss
Pulmonary	No change	Mild symptoms	Extertional dyspnoea	Dyspnoea at rest	Complete bed rest required
Alopecia	No change	Minimal hair loss	Moderate patchy alopecia	Complete alopecia but reversible	Non-reversible alopecia
Infection	None	Minor infection	Moderate infection	Major infection	Major infection with hypotension

* N = upper limit of normal value of population under study
