# Supplementary material for: BA08: An open-label, single-arm, non-randomised, phase 2 trial of cisplatin, methotrexate and vinblastine (CMV) for pure squamous cell cancer of the urinary tract
Source: PLoS One. 2019 Jan 16;14(1):e0210785. doi: 10.1371/journal.pone.0210785 (PMC6334943; doi:10.1371/journal.pone.0210785)
Supplement: S1 Text — (PDF) [file pone.0210785.s003.pdf]

S2 Appendix. Inclusion and exclusion criteria

Inclusion and exclusion criteria

Inclusion criteria:

1) Any patient with pure squamous cell carcinoma of the urinary tract in one of the following groups:
    a. initial presentation with T3-T4 disease
    b. pelvic relapse after radiotherapy or surgery
    c. nodal or metastatic disease
2) At least one site of disease must have been assessable for response by clinical examination or imaging, One or more of the sites below can be used to assess response:
    a. Primary bladder tumour
    Satisfactory measurements of the primary tumour if present and measureable, must have been made by bimanual examination after transurethral resection (TUR).
    b. Other primary tumours in the urinary tract
    Satisfactory measurements of the primary tumour in the urinary tract, if present and measureable, must have been made by clinical examination or appropriate imaging.
    c. Pelvic relapse after radiotherapy or surgery
    At least one indicator lesion must be measurable by clinical examination or appropriate imaging.
    d. Nodal or metastatic disease
    At least one indicator lesion must be measurable by clinical examination or appropriate imaging.
    N.B, Bone metastases cannot be used as an indicator lesion.
3) Calculated Glomerular filtration rate (GFR), calculated by the method of Cockcroft and Gault (1976) of >50ml/min. In patients with impaired renal function secondary to ureteric obstruction this may be relieved by ureteric stents or nephrostomies, and if the renal function then recovers the patient will be eligible.
4) WBC>3.5×10^9 l^{-1} and platelet count >100×10^9/l.
5) No previous systemic treatment with chemotherapy.
6) No concomitant or previous malignancy other than BCC of skin or carcinoma in-situ of the cervix.
7) Patients should be fit to tolerate CMV.

Exclusion criteria:

1) Patients with transitional cell carcinoma with squamous metaplasia, or other mixed tumours.
2) Co-existing illness (e.g. cardiac failure) which may compromise administration of CMV chemotherapy.
