# Supplementary material for: BA08: An open-label, single-arm, non-randomised, phase 2 trial of cisplatin, methotrexate and vinblastine (CMV) for pure squamous cell cancer of the urinary tract
Source: PLoS One. 2019 Jan 16;14(1):e0210785. doi: 10.1371/journal.pone.0210785 (PMC6334943; doi:10.1371/journal.pone.0210785)
Supplement: S2 Text — (PDF) [file pone.0210785.s004.pdf]

S3 Appendix: Definition of response

Definition of response

Complete response (CR)

The disappearance of all known malignant disease

Primary Bladder tumour

If the initial primary tumour was in the bladder, and cystectomy has not been performed, an assessment should be made of this primary tumour.

If the response of the primary bladder tumour is classified as complete response (CR) on bimanual examination and cystoscopy, a deep resection biopsy should be performed at the site of the original tumour and recorded as biopsy positive or negative, i.e.:

CR(B-)	Complete response on cystoscopic and bimanual examination. Biopsy negative.
CR(B+)	Complete response on cystoscopic and bimanual examination. Biopsy positive.
CR(Bo)	Complete response on cystoscopic and bimanual examination. Biopsy not done.

Partial response (PR)

At least 50% reduction of the sum of the products of the two largest perpendicular diameters of all lesions measured at registration. In addition there can be no appearance of new lesions nor progression of any lesion.

No change (NC)

Less than 50% reduction of the sum of the products of the two largest perpendicular diameters of all lesions measured at registration and no lesion measured at registration has shown a 25% increase in size.

Progressive disease

A 25% or more increase in the size of one or more lesions measured at registration, or the appearance of new lesions.
